# Supplementary material for: Multi-functional DNA nanostructures that puncture and remodel lipid membranes into hybrid materials
Source: Nat Commun. 2018 Apr 18;9:1521. doi: 10.1038/s41467-018-02905-w (PMC5906680; doi:10.1038/s41467-018-02905-w)
Supplement: Supplementary file 1 — Supplementary Information [file 41467_2018_2905_MOESM1_ESM.pdf]

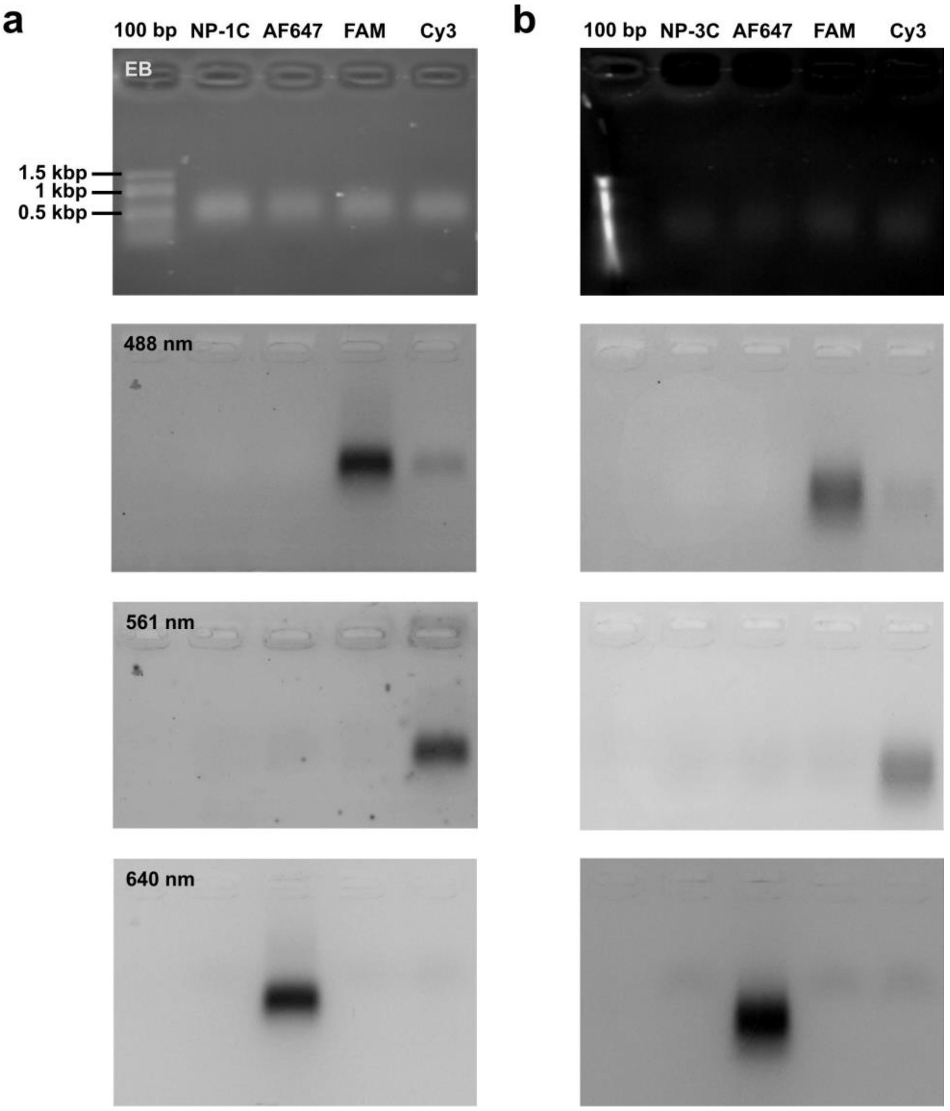

Supplementary Figure 1. 1.2 % SDS agarose gel electrophoretic analysis of DNA constructs. Assembly of DNA pores is complete and does not interfere with cholesterol and fluorescent dye modifications. (a) NP-1C with one cholesterol and (b) NP-3C with three cholesterol modified duplexes without and with the fluorescent dyes used in this study. Order for both gels (lane 1-5; from left to right): 100 bp ladder (New England Biolabs), no dye modification, AF647 modification, FAM modification and Cy3 modification. Top row ethidium bromide (EB) staining, followed by fluorescent images of green (FAM), orange (Cy3) and red (AF647) excitation respectively.

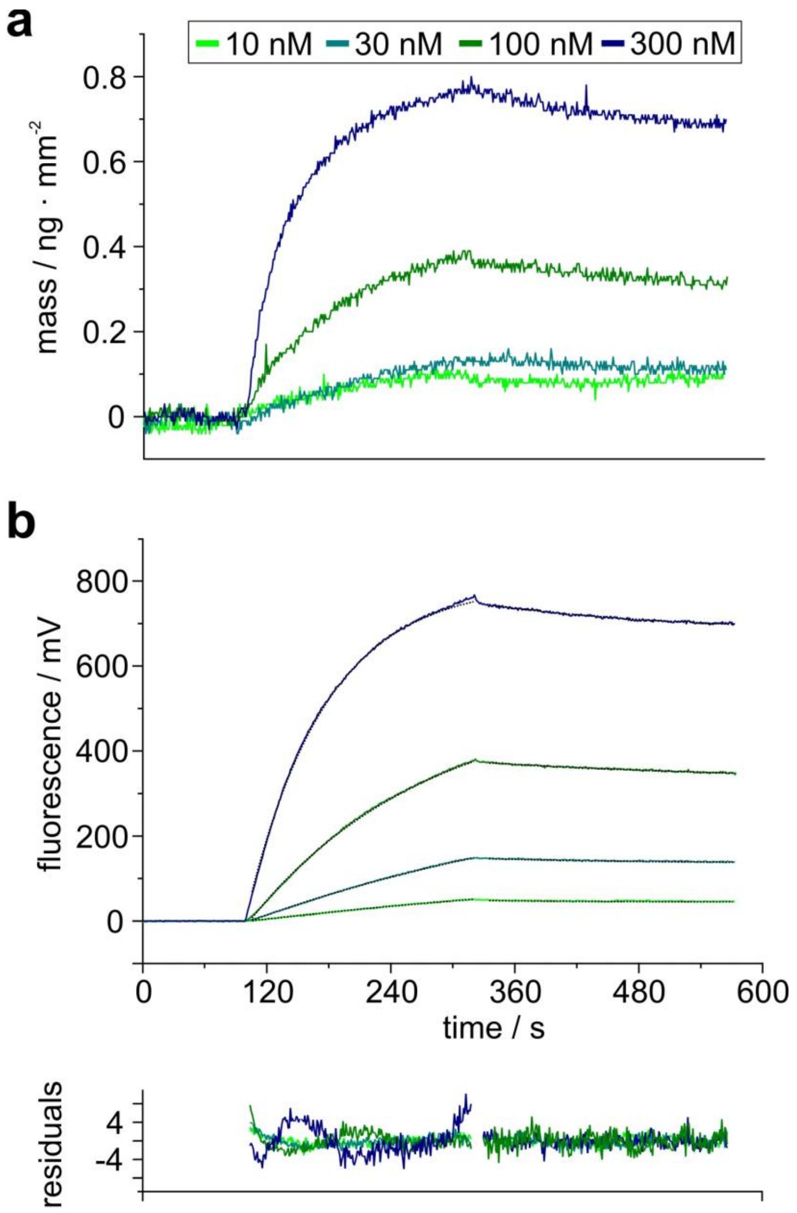

Supplementary Figure 2. Binding kinetics of ^{AF647}NP-3C at different concentrations. RIf (a) and TIRFS (b) curves obtained for concentration-dependent ^{AF647}NP-3C binding to PSM. In (b), an overlay with separate exponential fit curves assuming a 1:1 Langmuir model for determining the dissociation and association rate constants (top) and the residuals from the fit (bottom) are shown (dotted line).

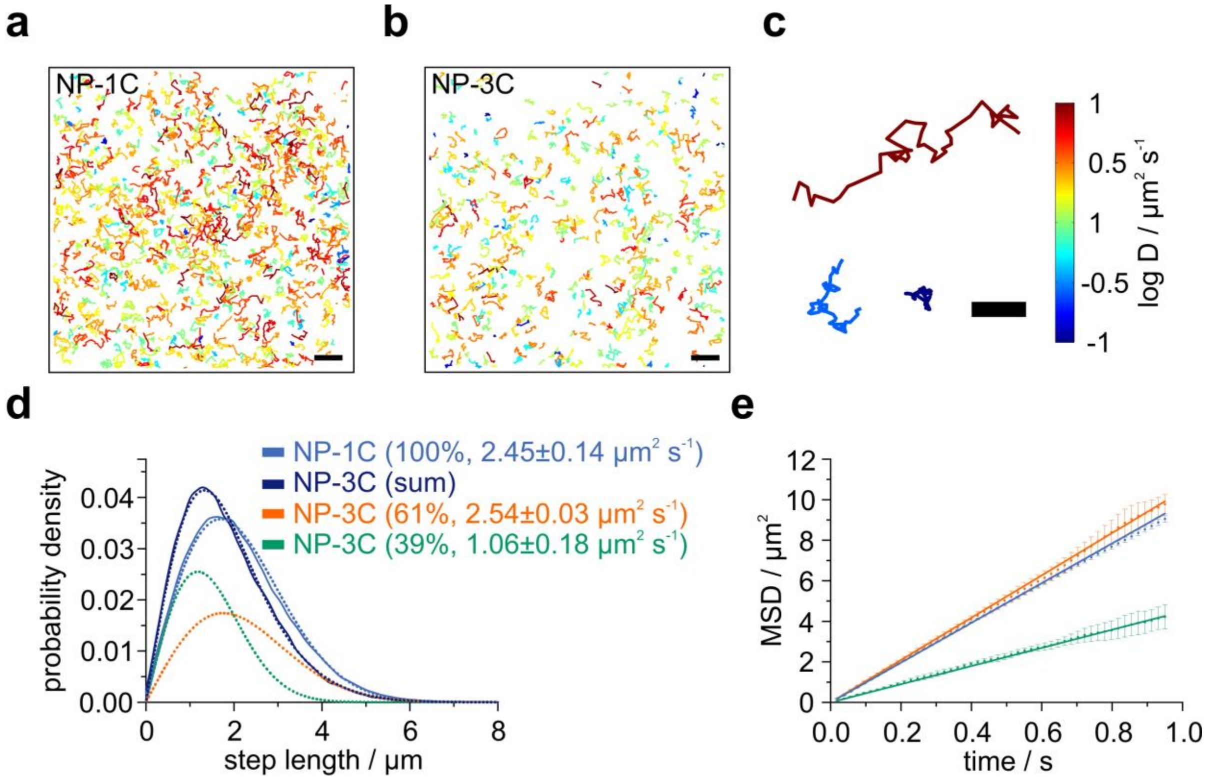

Supplementary Figure 3. Diffusion properties of mobile ^{AF647}NP-1C and ^{AF647}NP-3C bound to PSM. (a, b) Color-coded trajectories of mobile ^{AF647}NP-1C (a) and ^{AF647}NP-3C pores (b) at the same time point (t= 0 min). The scale bars correspond to 5 µm. (c) Representative trajectories covering the full range of observed diffusion coefficients each of 50 frames length. The black scale bar corresponds to 2 µm. (d) Empiric step length distributions over 30 frames for ^{AF647}NP-1C and ^{AF647}NP-3C (solid lines) and component fits (dotted lines). While for ^{AF647}NP-1C a single population with a diffusion coefficient D = 2.45 ± 0.03 µm^2 s^{-1} (light blue) was sufficient, a two-population model for ^{AF647}NP-3C was used. 39% of ^{AF647}NP-3C showed a decreased mobility of 1.06 ± 0.18 µm^2 s^{-1} (green), while the majority (61%) showed fast diffusion of 2.54 ± 0.14 µm^2 s^{-1} (orange). (e) Mean square displacement (MSD) analysis of ^{AF647}NP-1C and ^{AF647}NP-3C over prolonged time (0.95 s – 50 frames) showing linear long-range Brownian motion. Diffusion coefficients derived from the slope are 2.58 ± 0.05 µm^2 s^{-1} for ^{AF647}NP-1C (light blue) and 1.18 ± 0.03 µm^2 s^{-1} (green) and 2.75 ± 0.06 µm^2 s^{-1} (orange) for ^{AF647}NP-3C, respectively. All diffusion coefficients are mean values and s.d. from a minimum of 3 separate experiments with several thousand trajectories per experiment.

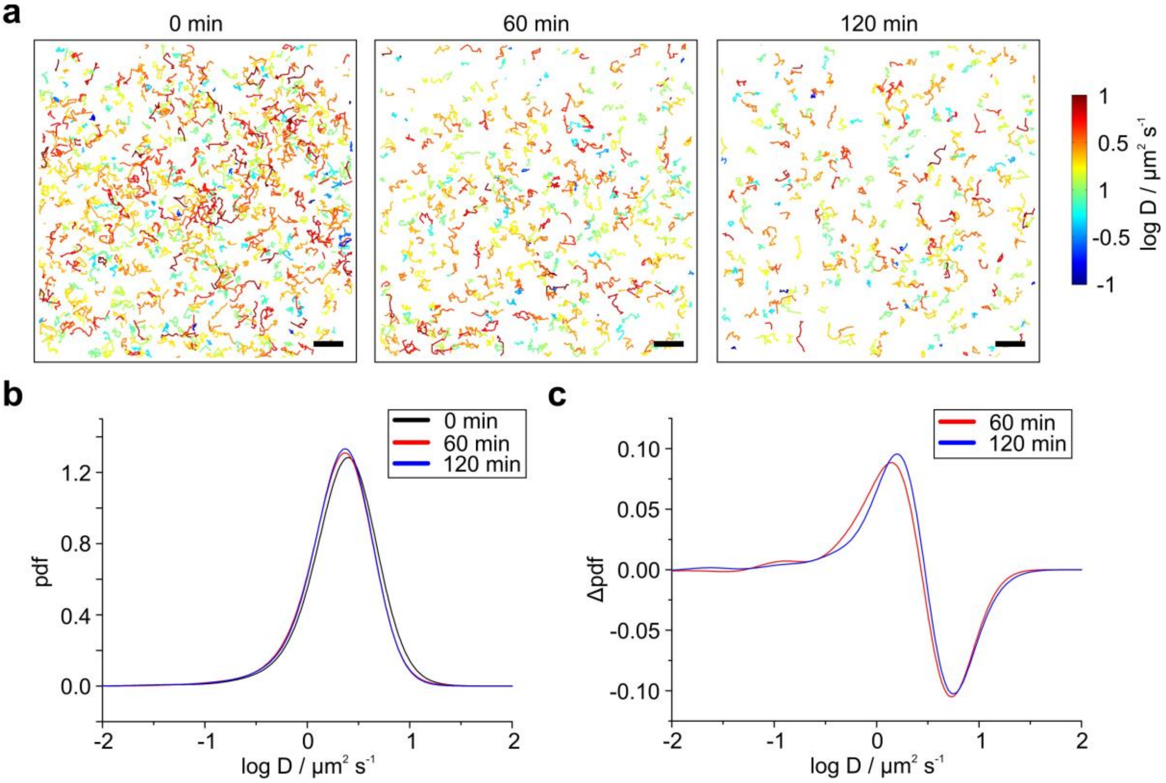

Supplementary Figure 4. Diffusion properties of PSM-anchored ^{AF647}NP-1C. (a) Color-coded trajectories of mobile ^{AF647}NP-1C pores at the indicated time points with fast moving species in red and slow moving pores depicted as blue according to the color scheme. The scale bars correspond to 5 µm. b) Probability density function (pdf) of the diffusion coefficient of ^{AF647}NP-1C at different time points. c) Difference in the probability density function (Δpdf) between later time points and t = 0 min.

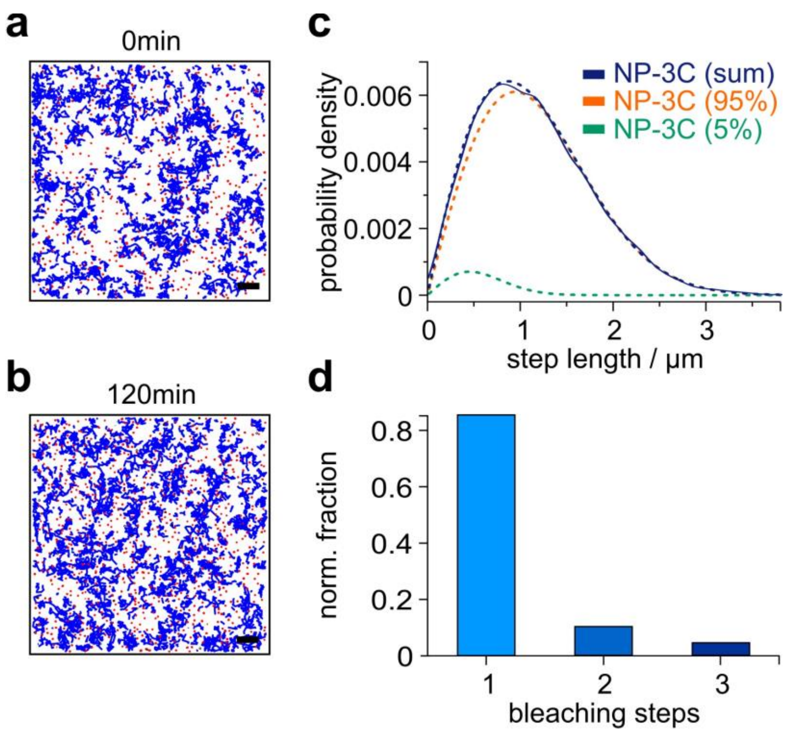

Supplementary Figure 5. Supported lipid bilayers (SLBs) prevent NP membrane insertion and dynamic clustering. (a, b) Trajectory maps from 60 consecutive frames of mobile (blue) and immobile (red) ^{AF647}NP-3C directly after binding to the SLB (0 min, a) and two hours later (120 min, b). (c) Step length analysis of the mobile fraction of ^{AF647}NP-3C on SLBs revealing a major fraction of 95% undergoing fast diffusion (orange: 2.67 ± 0.05 µm^2 s^{-1}) and a minor fraction of 5% undergoing slower diffusion (green: 1.28 ± 0.46 µm^2 s^{-1}). Data depicts the mean and s.d. of n=3 experiments. (d) STaSI bleaching step analysis of ^{AF647}NP-3C after 2 hours of incubation with the SLB (n=4 with 900 detected clusters). Scale bars: 5 µm.

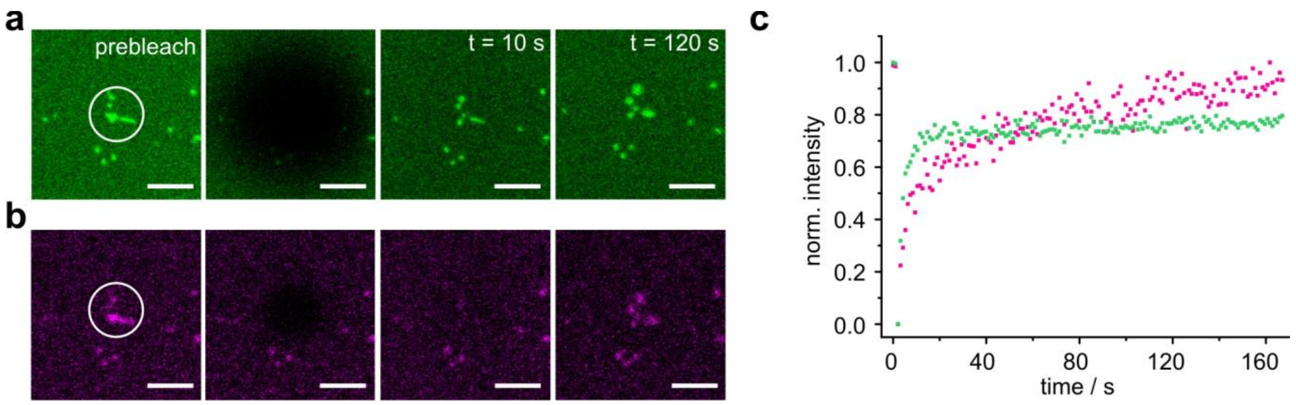

Supplementary Figure 6. Fluorescence recovery after photobleaching (FRAP) of DNA-NP induced membrane protrusions and associated DNA NPs. (a, b) ^{OG488}DHPE (a) and ^{AF647}NP-3C (b) channels before bleaching a circular area (white circle) with high laser power and the same ROI imaged at different time points after photobleaching. (c) Fluorescence intensity in the photobleached area over time. Sale bars: 5 µm.

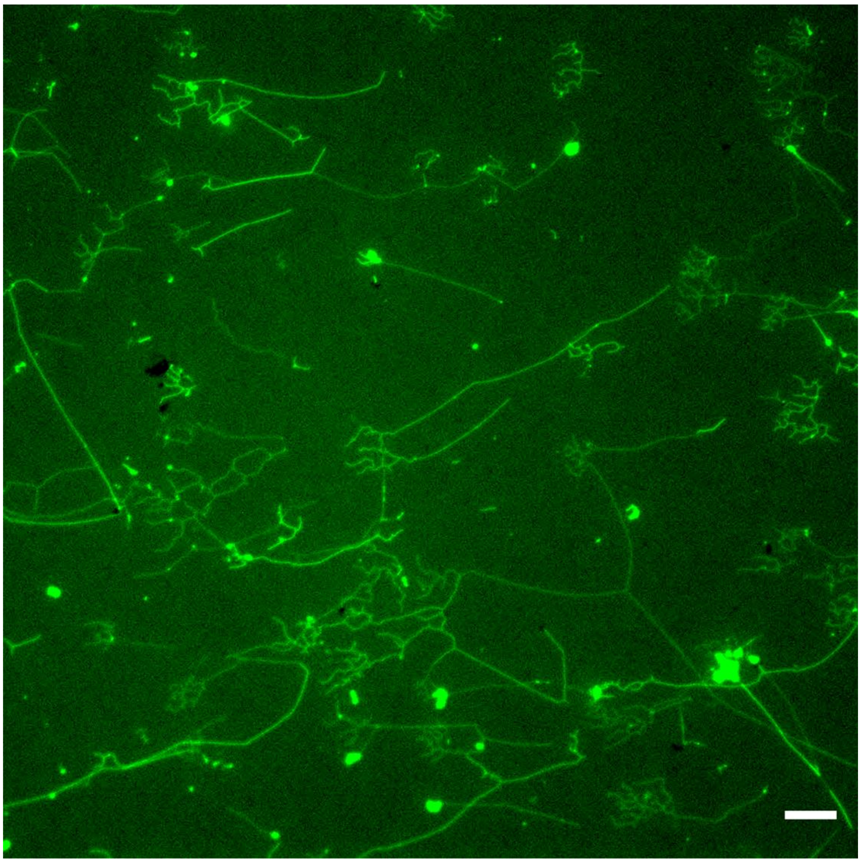

Supplementary Figure 7. High density of lipid nanotubes induced by NP-3C on PSMs. TIRF image of ^{OG488}DHPE-stained lipid nanotubes on PSM that were loaded with NP-3C prior to vesicle fusion. Scale bar: 10 µm.

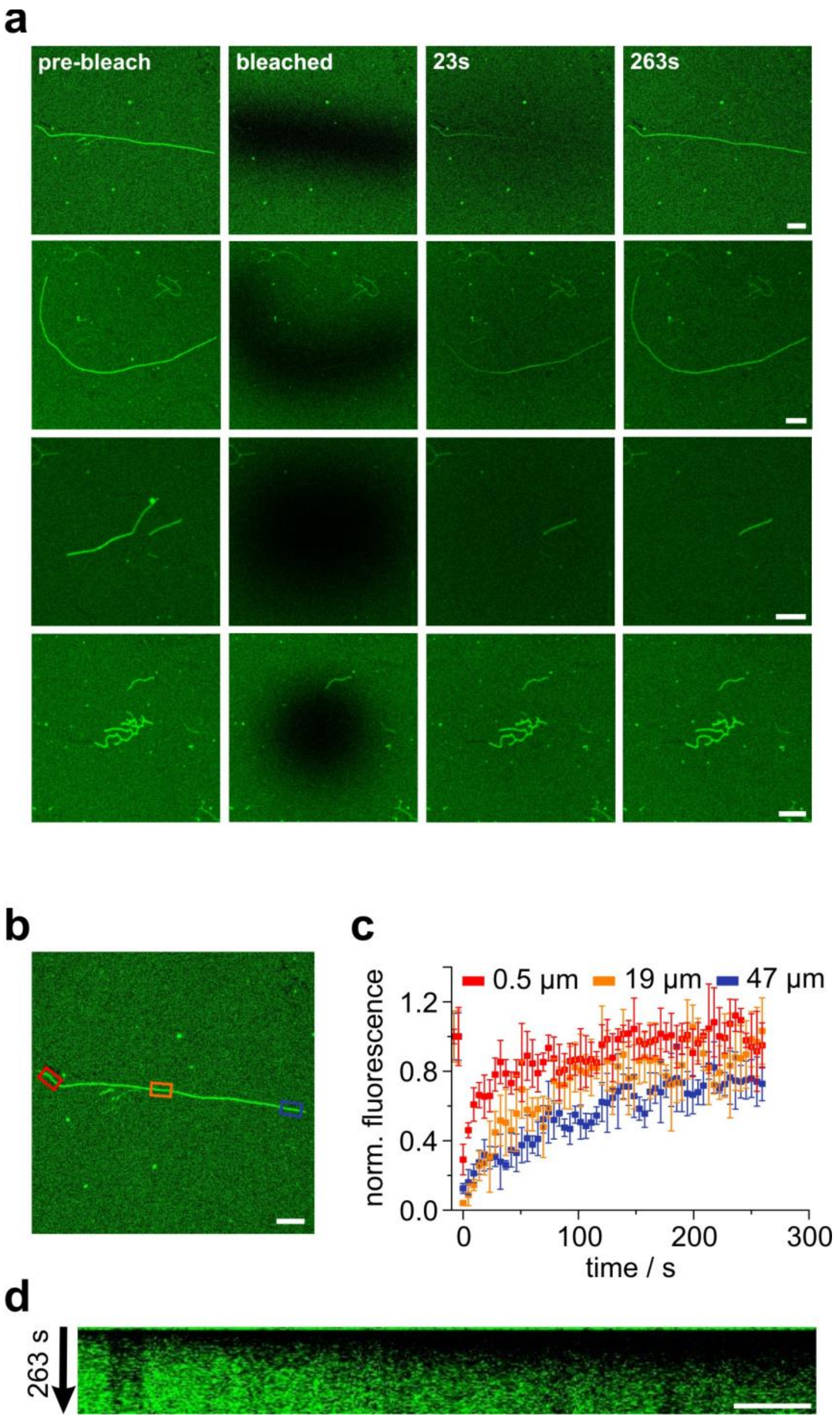

Supplementary Figure 8. Lipid nanotubes are predominantly connected to the underlying membrane. (a) Representative confocal fluorescence images of ^{OG488}DHPE doped PSMs with lipid nanotubes. (b, c) From the experiment shown in the top row of panel a, the kinetics of fluorescence recovery within different ROIs depicted in panel b was plotted (c). (d) Kymograph of the fluorescence along the nanotube seen in (b). Scale bar: 5 µm in all panels.

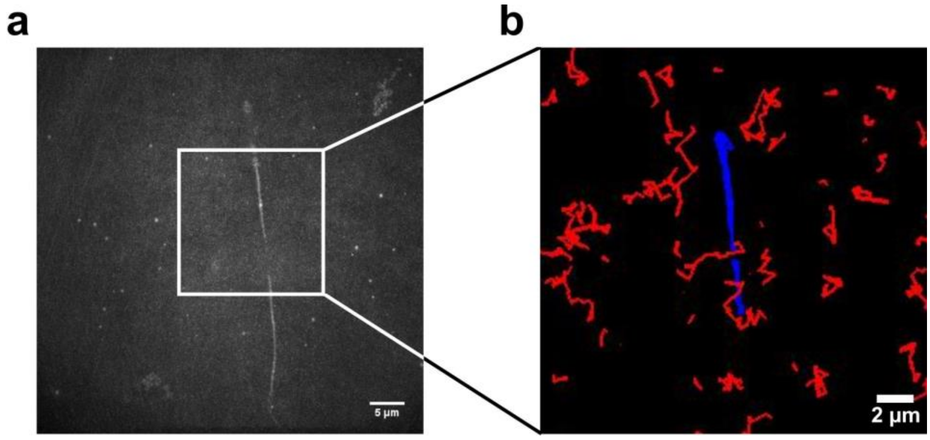

Supplementary Figure 9. Lipid diffusion in PSM and lipid tubes. (a) Maximum intensity projection of 100 consecutive frames of DiD after incubation of the PSM with ^{Cy3}NP-3C. (b) Overlay of tracked confined mobility signals of DiD (blue - binning of 1500 frames) with tracked mobile DiD molecules (blue).

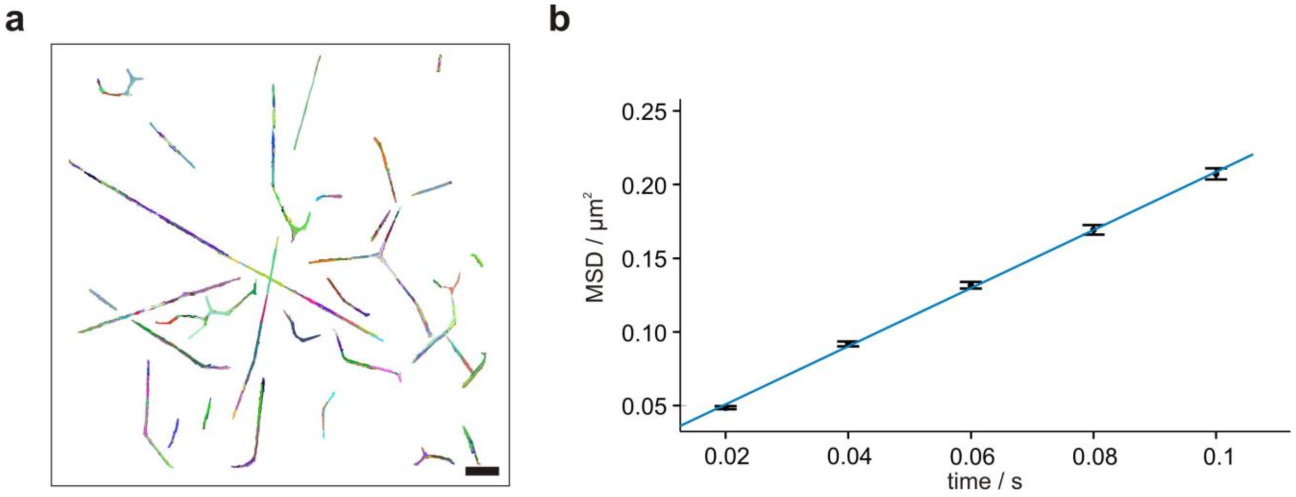

Supplementary igure 10. Diffusional analysis of lipid tube associated NP-3C. (a) Pooled trajectories (out of 20 experiments) of ^{Dy647}NP-3C diffusing in lipid nanotubes. Scale bar: 2 µm. (b) Mean square displacement analysis of the first 5 steps of nanotube-restricted diffusion visible in a) assuming one-dimensional diffusion, yielding a diffusion coefficient D of 0.99 ± 0.03 $\mu m^2 \cdot s^{-1}$ (mean + s.e.m. from pooled trajectories of n=15 experiments).

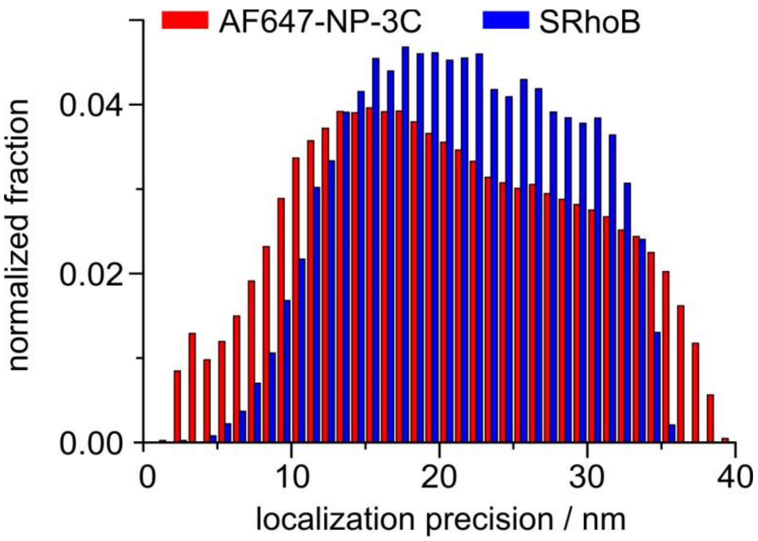

Supplementary Figure 11. Precision of single molecule localization. Distribution of localization precisions obtained for individual ^{AF647}NP-3C and sulforhodamine B (SRhoB) in dual color experiments. Median values are 19.8 nm for AF647 and 25.4 nm for SRhoB calculated from 171123 and 30903 individual localizations, respectively.

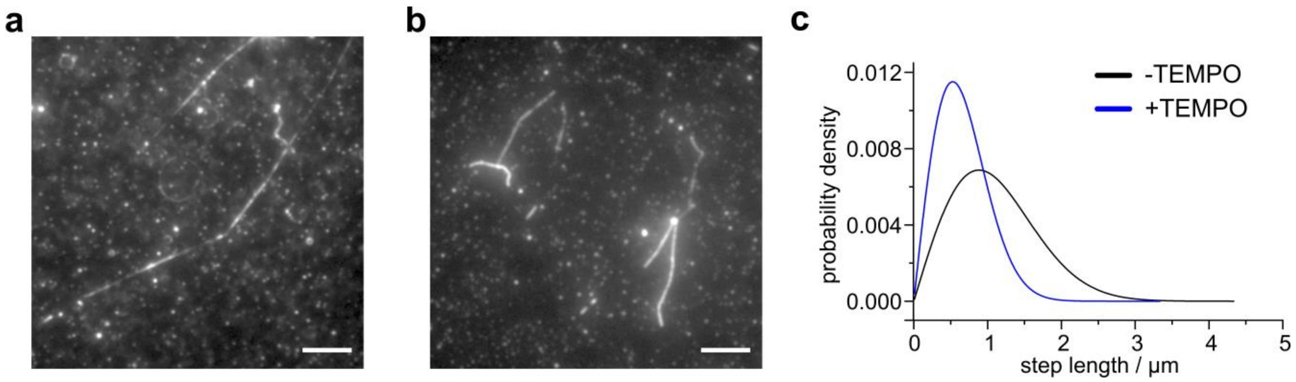

Supplementary Figure 12. Accessibility of ^{FAM}NP-3C bound to lipid nanotubes. (a, b) Maximum intensity projection of 150 frames of ^{FAM}NP-3C before (a) and after (b) addition of 50 mM TEMPO. c) Step lengths histograms of mobile ^{FAM}NP-3C determined with or without TEMPO for 10 consecutive frames. Scale bars: 2 µm.

Supplementary Table 1. IDs, chemical modifications and sequences of DNA oligonucleotides used to prepare DNA nanopores

ID	Sequence 5' → 3'
1	AGCGAACGTGGATTTTGTCCGACATCGGCAAGCTCCCTTTTTCGACTATT
2	CCGATGTCGGACTTTTACACGATCTTCGCCTGCTGGGTTTTGGGAGCTTG
3	CGAAGATCGTGTTTTTCCACAGTTGATTGCCCTTCACTTTTCCCAGCAGG
4	AATCAACTGTGGTTTTTCTCACTGGTGATTAGAATGCTTTTGTGAAGGGC
5	TCACCAGTGAGATTTTTGTCGTACCAGGTGCATGGATTTTTGCATTCTAA
6	CCTGGTACGACATTTTTCCACGTTCGCTAATAGTCGATTTTATCCATGCA
1(Chol)	Sequence of 1 carrying tri(ethylene glycol) cholesterol at the 3' terminus
3(Chol)	Sequence of 3 carrying tri(ethylene glycol) cholesterol at the 3' terminus
4(Chol)	Sequence of 4 carrying tri(ethylene glycol) cholesterol at the 3' terminus
5(Chol)	Sequence of 5 carrying tri(ethylene glycol) cholesterol at the 3' terminus
2(AF647)	Sequence of 2 carrying AF647 at the 5' terminus
2(Cy3)	Sequence of 2 carrying Cy3 at the 5' terminus
2(FAM)	Sequence of 2 carrying 6-fluorescein NHS ester at the 5' terminus

Supplementary Table 2. Name and composition of the assembled nanopores

Nanopore	Oligonucleotide composition
NP-0C	1, 2(AF647), 3, 4, 5, 6
NP-1C	1, 2(AF647), 3, 4(Chol), 5, 6
NP-3C	1(Chol), 2(AF647/Cy3/FAM), 3(Chol), 4, 5(Chol), 6

Supplementary Table 3. UV melting points of modified DNA NPs

	NP			^{AF647}NP			^{Cy3}NP		
	0C	1C	3C	0C	1C	3C	0C	1C	3C
T_m (°C)	51.0	51.0	51.0	51.3	51.2	51.3	50.5	51.5	51.5
T_m SUV (°C)	n.d.	52.0	60.0	n.d.	52.3	59.2	n.d.	52.5	60.6
net change (°C)		1.0	9.0		1.1	8.0		0.9	9.1

Supplementary Table 4. Kinetic constants of binding of ^{AF647}NP-3C and ^{AF647}NP-1C to PSMs measured by TIRFS-RIf

	^{AF647}NP-3C				^{AF647}NP-1C
c (nM)	10	30	100	300	100
k_a (10^4 $M^{-1} \cdot s^{-1}$)	15 ± 2	17 ± 2	7.4 ± 0.3	5.1 ± 0.3	48 ± 2
k_d (10^{-4} s^{-1})	4.1 ± 0.2	4.1 ± 0.4	3.6 ± 0.1	2.9 ± 0.1	58 ± 3
K_D (nM)	3	2	5	6	12
