# Supplementary material for: Multi-functional DNA nanostructures that puncture and remodel lipid membranes into hybrid materials
Source: Nat Commun. 2018 Apr 18;9:1521. doi: 10.1038/s41467-018-02905-w (PMC5906680; doi:10.1038/s41467-018-02905-w)
Supplement: Supplementary file 3 — Description of Additional Supplementary [file 41467_2018_2905_MOESM3_ESM.docx]

**Description of Additional Supplementary Files**

File Name: Supplementary Movie 1

Description: Binding of DNA NPs to PSMs as detected by single molecule imaging. PSMs were incubated with 5 pM ^AF647^NP-0C (left), ^AF647^NP-1C (center) and ^AF647^NP-3C (right), respectively and immediately imaged by TIRFM at 53 fps.

File Name: Supplementary Movie 2

Description: Time-dependent change in mobility of NP-3C bound to PSM. PSMs were incubated with 5 pM ^AF647^NP-3C and imaged by TIRFM at 53 fps directly after addition (left) and 60 min later (right).

File Name: Supplementary Movie 3

Description: Time-dependent change in mobility of NP-1C bound to PSM. PSMs were incubated with 5 pM ^AF647^NP-1C and imaged by TIRFM at 53 fps directly after addition (left) as well as 60 min (center) and 120 min later (right).

File Name: Supplementary Movie 4

Description: Analysis of cluster sizes by single molecule photobleaching. PSMs were incubated with two different ^AF647^NP-3C concentrations, resulting in different densities of immobile pores after 60 min. The immobile pores were subsequently illuminated at 50 fps by TIRFM to observe intensity decreases by single-step photobleaching.

File Name: Supplementary Movie 5

Description: Enrichment of DNA NPs in membrane protrusions. PSMs with spare lipid material were briefly incubated with 5 pM ^AF647^NP-3C and after 10 min, a manual z-scan out of the membrane plane to the top of the PSM was acquired by TIRFM at 33 fps.

File Name: Supplementary Movie 6

Description: Dynamics of membrane protrusion formation induced by DNA NPs. ^OG488^DHPE doped PSMs were incubated with 5 nM ^AF647^NP-3C and after 10 min, both lipid (green) and ^AF647^NP-3C (magenta) fluorescence channels were imaged at 1 fps by cLSM.

File Name: Supplementary Movie 7

Description: One-dimensional diffusion of NP-3C in lipid nanotubes. PSMs were incubated with 5 pM ^AF647^NP-3C and shortly after, regions with characteristic one-dimensional diffusion (overlay of red trajectories) were imaged by TIRFM at 91 fps.

File Name: Supplementary Movie 8

Description: Connection of lipid nanotubes with the underlying PSM explored by FRAP. ^OG488^DHPE doped PSMs were incubated with 5 nM ^AF647^NP-3C for 10 min and subsequently, formed isolated lipid nanotubes were selectively bleached and fluorescence recovery was imaged by cLSM at 1.2 fps.

File Name: Supplementary Movie 9

Description: Connection of lipid nanotubes with the underlying PSM explored by single molecule tracking. PSMs were incubated with 5 pM ^AF647^NP-3C and shortly after, a mobile DNA NP (blue trajectory) diffusing in the PSM in close proximity to a lipid nanotube (red overlay) was imaged by TIRFM at 40 fps.

File Name: Supplementary Movie 10

Description: Dual color TALM imaging of DNA NP and SRhoB in lipid nanotubes. PSMs were incubated with 5 pM of ^AF647^NP-3C (green) for 10 min, followed by a short incubation with soluble SRhoB (magenta) and subsequent dual color TIRFM at 40 fps. Shown is a raw data overlay of both channels (left) with a processed accumulative TALM image (right).

File Name: Supplementary Movie 11

Description: Collisions of individual DNA NPs in lipid nanotubes. PSMs were co-incubated with 5 pM of ^AF647^NP-3C (green) and ^Cy3^NP-3C (magenta). DNA NP diffusion in the formed lipid nanotubes was imaged by TIRFM at 91 fps (playback at 10% of real-time). Shown are the raw data (left) and the single-molecule localizations (right) with a kymograph analysis along the nanotube (white box) shown at the bottom.
